# Development of a Population-Based Newborn Screening Method for Severe Combined Immunodeficiency in Manitoba, Canada

**DOI:** 10.3390/ijns4020019

**Published:** 2018-06-19

**Authors:** J. Robert Thompson, Cheryl R. Greenberg, Andrew Dick, Olga Jilkina, Luvinia Kwan, Tamar S. Rubin, Teresa Zelinski, Marlis L. Schroeder, Paul Van Caeseele

**Affiliations:** 1 Cadham Provincial Laboratory, Winnipeg, MB R3E 3J7, Canada; 2 Department of Pediatrics and Child Health, Max Rady College of Medicine, Rady Faculty of Health Sciences, University of Manitoba, 546-715 McDermot Ave, Winnipeg, MB R3E 3R4, Canada; 3 CancerCare Manitoba, Winnipeg, MB R3E 0V9, Canada

**Keywords:** newborn screening, SCID, TREC

## Abstract

The incidence of Severe Combined Immunodeficiency (SCID) in Manitoba, (1/15,000), is at least three to four times higher than the national average and that reported from other jurisdictions. It is overrepresented in two population groups: Mennonites (*ZAP70* founder mutation) and First Nations of Northern Cree ancestry (*IKBKB* founder mutation). We have previously demonstrated that in these two populations the most widely utilized T-cell receptor excision circle (TREC) assay is an ineffective newborn screening test to detect SCID as these patients have normal numbers of mature T-cells. We have developed a semi-automated, closed tube, high resolution DNA melting procedure to simultaneously genotype both of these mutations from the same newborn blood spot DNA extract used for the TREC assay. Parallel analysis of all newborn screening specimens utilizing both TREC analysis and the high-resolution DNA procedure should provide as complete ascertainment as possible of SCID in the Manitoba population.

## 1. Introduction

Severe Combined Immunodeficiency (SCID) is the most profound form of the primary immunodeficiency diseases (PID) and is characterized by the lack of a functioning immune system. Infants born with SCID are normal at birth but invariably develop multiple severe infections which usually prove fatal in the first year of life. Treatment by hematopoietic stem cell transplantation early in life is associated with a good outcome and the highest probability of long-term survival [1]. Pre-symptomatic diagnosis and early intervention greatly improve the outcome of children with this condition with the potential to save lives and prevent suffering. In March 2015, the Federal US Committee on Newborn Screening recommended the addition of Severe Combined Immunodeficiency (SCID) to the routine newborn screening panel and now is offered in over 40 States in the United States [2,3]. Since then several countries including Israel [4], Taiwan [5] and one Canadian province of Ontario [6] have introduced newborn screening for SCID. Many others are conducting pilot projects or have proposals being considered [7,8,9,10]. The most widely used newborn screening test for SCID is the quantification of T-cell receptor excision circles (TREC) [11].

Canadian surveillance studies have found that SCID incidence is 3-fold higher in the province of Manitoba compared to the rest of the country [12,13]. In a retrospective study [13] of the 18 children diagnosed with SCID and other PID in Manitoba between 1992 and 2010, we demonstrated that more than half of these affected children would not have been identified on newborn screening by TREC analysis. The children who would have not been ascertained by TREC analysis belong to two separate population groups, each with a founder mutation that contributes to the disproportionately high frequency of SCID in Manitoba. One group is of Mennonite descent and has zeta chain-associated protein kinase (ZAP70) deficiency [14] and the second group is of First Nations Northern Cree ancestry with inhibitor of kappa light polypeptide gene enhancer in B-cells, kinase beta (IKBKB) deficiency [15]. The Mennonite ZAP70 deficiency seen in our population results from a homozygous G>A substitution in the acceptor splice site of intron 12 of *ZAP70* (c.1624−11G>A) abolishing the usual acceptor splice site and creating a new acceptor splice sequence upstream. This results in the insertion of 9 nucleotides in the mRNA and 3 additional amino acids in the protein product, inactivating the kinase. The Northern Cree *IKBKB* mutation results from the homozygous insertion of a G at nucleotide 1292 in exon 13 of the *IKBKB* gene (c.1292dupG) causing a frameshift mutation and a premature stop codon with complete loss of kinase function of the IKBKB protein. The latter is integral to the nf-Kb pathway by phosphorylating inhibitors of Kb, thereby impacting T and B cell receptors [15]. A semi-automated method involving closed-tube (homogeneous) high-resolution melting analysis for the simultaneous genotyping of these two founder mutations was developed and run in parallel with the quantification of TREC on each newborn dried filter paper blood spot (DBS) collected as part of routine newborn screening. We now report details of this methodology and its validation and emphasize the importance of ensuring that ascertainment is as complete as possible when introducing universal newborn screening for SCID.

## 2. Materials and Methods

### 2.1. Patient Studies

DNA was extracted from 46 DBS retrieved from patients known to be at risk for ZAP70 deficiency and IKBKB deficiency, genotyped in previous studies as *n* = 3 *ZAP70* or *n* = 2 *IKBKB* homozygous affected; *n* = 6 *ZAP70* or *n* = 14 *IKBKB* heterozygous; and *n* = 1 *ZAP70* or *n* = 20 *IKBKB* homozygous normal with respect to the *ZAP70* and *IKBKB* mutations [13,14,15]. The 46 DNA eluates were then amplified and analyzed by the high-resolution melting technique as described below and assigned a genotype. The amplicons assigned homozygous affected, heterozygous or homozygous normal genotypes were then compared to the Sanger sequencing results generated from the 46 original DBS to validate the genotype assignments from the high-resolution melting technique. The study was conducted in accordance with the Declaration of Helsinki and was approved on June 19, 2013 by the Research Ethics Board of the University of Manitoba (H2013:180).

### 2.2. High Resolution Melting DNA Studies

A quantitative real-time PCR (qPCR) assay was designed to make use of the high-resolution DNA melting curves by exploiting the difference in the melt temperature (Tm) of amplicons resulting from the single nucleotide substitutions in *ZAP70* and *IKBKB* from individuals with different genotypes. Primers were designed to ensure similar melt temperatures (Tm) for forward and reverse primers and short amplicon product sizes of less than 100 bp, based on the rationale that the shorter the amplicon, the greater the effect of a single base change on the melting temperature of the product. The primer sequences and their Tms were as follows (Table 1).

DNA was extracted from each DBS using a 3 mm spot punched by an Eppendorf DBS puncher and lysed using Qiagen Biosprint 96 lysis buffers (QIAGEN Sciences, Germantown, MD, USA) containing Proteinase K at 56 °C for 60 min while shaking at 900 rpm. DNA was extracted from the samples over 40 min using the Biomerieux Easymag Nuclisenssystem with magnetic silica beads (Biomerieux Inc. Canada, St. Laurent, QC, Canada) to produce a 25 µL eluate of purified DNA. Eluates were stored at 4 °C until analysis.

Amplification of 5 µL aliquots was performed using the BioradCFX96 Thermocycler C1000 Touch System (Bio-Rad Laboratories (Canada) Ltd., Mississauga, ON, Canada) using Biorad SsoFast EvaGreen Supermix (Biorad Cat. No. 1725201). Reaction mixtures (total volume 20 µL) were prepared by combining 10 µL SsoFast EvaGreen Supermix, 0.2 µL of each 20 µM forward and reverse IKBKB primers, 0.2 µL of each 10 µM forward and reverse ZAP70 primers, 5 µL extracted DNA eluate from patients and controls and 4.2 µL DNase/RNase free water to bring final reaction volume to 20 µL. Product amplification was monitored by the increase in fluorescent intensity using the following cycling parameters. Initial DNA denaturation at 98 °C for 2 min (to complete the activation of hot start polymerase) was followed by denaturation at 98 °C for 5 s and annealing/extension at 58 °C for 10 s. This was repeated for 39 cycles with a plate read at the end of each cycle followed by a final denaturation at 95 °C for 30 s and final anneal/extension at 70 °C for 30 s. Melt curves were then generated between 75–95 °C by incrementally increasing the temperature in steps of 0.2 °C for 10 s per step followed by plate read.

The melt curve profile data were generated by the CFX Manager software (Bio-Rad Precision Melt Analysis Software Version 1.25 (1.2.131.1030), Mississauga, ON, Canada) based on measurement of the decrease in fluorescent signal at each incremental step of double stranded amplicon denaturation. The amplicon melting temperature was defined at the point at which there was a decrease of 50% in fluorescent signal intensity. The Precision Melt Analysis software interpreted the melt curve data following signal strength normalization due to the variable intensity of the starting signal between specimens.

## 3. Results

**Figure 1:**
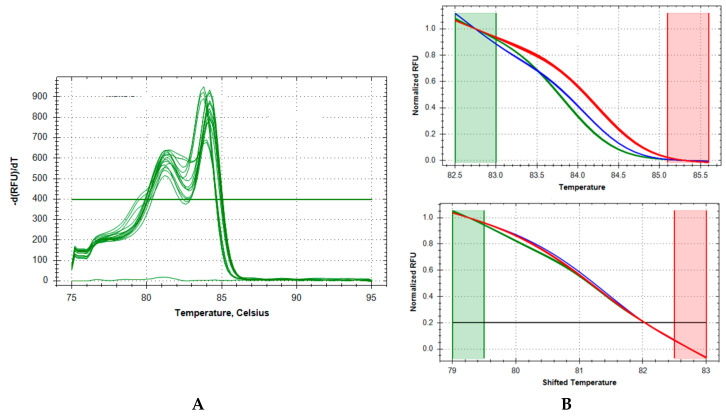
Negative First Regression Adjusted Raw Fluorescent Data of Amplified Products. In Panel A the peaks of ZAP70* and IKBKB^#^ represent the melting temperatures of all the ZAP70 and IKBKB amplicons where there is 50% loss of fluorescent signal intensity, that is, the point at which one half of the double stranded amplicon DNA is denatured. The rate of change in fluorescence as the temperature rises is determined by plotting the negative first regression of relative fluorescence (RFU) vs. Temperature (−d(RFU)/dT) on the y-axis. Panel B shows the results when the Precision Melt Analysis software normalized the raw fluorescent data (**A**) and set pre- and post-melt signals to relative values of 1.0 to 0. The normalized melt curves produced were distinct for the ZAP70 amplicons (**Top B**) and the IKBKB amplicons (**Bottom B**).

Figure 1 illustrates the raw fluorescence data of the Precision Melt Analysis adjusted by the negative first derivative visualizing the melting temperatures of the ZAP70 and IKBKB amplicons (A) and the normalized melt curves (B). In Panel A the melting temperatures of the amplicons were assigned at the point where there is a 50% loss of fluorescent signal intensity, that is, the point where one half of the double stranded amplicon is denatured. Panel B shows the results when the Precision Melt Analysis software normalized the raw fluorescent data and set pre- and post-melt signals to relative values of 1.0 to 0. The normalized melt curves produced clear differentiation between the ZAP70 (Top B) and IKBKB (Bottom B) amplicons.

**Figure 2:**
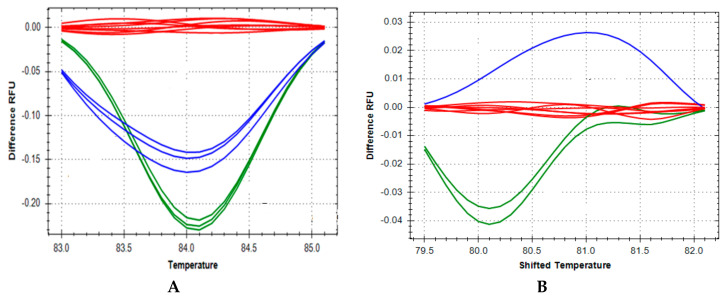
Representative Temperature Shifted Difference Curves. The temperature shifted difference curves further resolve clusters of the same genotype and clearly differentiate the *ZAP70* homozygous affected (green line), heterozygous (blue line) and wild type (red line) genotypes (**A**) and the *IKBKB* homozygous affected (blue line), heterozygous (green line) and wild type (red line) genotypes (**B**).

The temperature shifted difference curves further resolved and clearly differentiated the *ZAP70* and *IKBKB* homozygous affected, heterozygous and wild type genotypes respectively (Figure 2A,B). The *ZAP70* and *IKBKB* genotype assignments from the 46 DBS were validated by Sanger sequencing of DNA extracted from the original DBS samples. The high resolution melting technique used in the DNA studies also assigned a genotype of homozygous normal to 60 unrelated, random, age-matched, unaffected control samples with 100% accuracy.

## 4. Discussion

Although TREC screening should identify 100% of T-cell deficient forms of SCID and PID, we have previously shown that it will not identify the majority of T-cell positive forms of SCID and PID prevalent in our population due to our 2 known founder mutations. There are ~16,000 births annually in the province of Manitoba. One baby with the *ZAP70* Mennonite founder mutation and one baby with the *IKBKB* Northern Cree founder mutation are diagnosed, on average, yearly. These babies become symptomatic and either die of their disease or are referred for bone marrow transplantation. Without screening at birth, these babies with SCID have very prolonged and complicated hospitalizations and, if they survive to transplant, have very stormy post-transplant courses. The cost of an early successful transplant in an infant ascertained on newborn screening in our province is less than the costs of prolonged hospitalization and morbidity suffered by managing a patient with SCID after a late diagnosis. The actual cost of the materials for DNA extraction followed by quantitative PCR for TREC/RNAse P and high resolution melt analysis for both founder mutations is ~$15.00 Canadian per test.

Implementation of the high-resolution DNA melting analysis described in this manuscript for the determination of the specific *ZAP70* and *IKBKB* genotypes in all patients run in parallel with TREC/RNaseP quantification should provide as complete ascertainment as possible of SCID in the newborn population of Manitoba. We realize that other mutations in *ZAP70* and *IKBKB* in our population and in other populations will not be identified by this specific high resolution melt analysis and is a limitation of our approach. Other methodologies such as Next Generation Sequencing (NGS) could be considered but at the present time are not feasible as a first-tier clinical test for universal newborn screening in our province [16]. Thus, combining TRECS with high resolution melt for these 2 founder mutations as we describe will have the best sensitivity and the lowest likelihood of false negatives in universal neonatal screening for SCID in Manitoba. This methodology could also be implemented in other Canadian provinces where these 2 founder mutations are also present in their Mennonite [14] and Indigenous communities [15] or can be adapted to other populations with their own specific founder mutations when there are false negative results with TRECS alone. Our approach is also conceptually similar to that described recently by Al- Mousa et al. [7] using a Targeted-NGS [T-NGS] Primary Immunodeficiency panel in their Saudi population with a very high frequency of SCID. However, their approach is different from ours as T-NGS is a second tier mutation screening tool on DBS from Saudi newborns ascertained by low TREC assays. Both Biggs et al. [17] and Rechavi et al. [4] discuss the challenge of a TRECS-based assay alone for neonatal SCID detection, its fundamental inability to detect immunodeficiencies that result from T-cell dysfunction without T-cell lymphopenia and the need to be aware of these T-cell positive SCID variants that will be missed by TRECS- methodology alone. With our combined methods we believe we will significantly reduce the number of false negatives in our Manitoba population than if we used TRECS alone. The methodology is semi-automated, scalable and reproducible allowing for high throughput, rapid universal newborn screening for SCID and for those particular conditions prevalent in our province, a programme we hope to implement in the coming year.

## Figures and Tables

**Table 1:** Primer sequences and Tms for PCR amplification.

Name	Sequence	Length	Tm
IKBKB Forward Primer	5′-AGG AAT CTC GCC TTC TTC C-3′	19	56.62
IKBKB Reverse Primer	5′-CTG GAT GCT GTG CCA GAC-3′	18	58.10
ZAP70 Forward Primer	5′-TGA GGA GGA GGA CAC TGG-3′	18	57.13
ZAP70 Reverse Primer	5′-TTG CCC TGC TCG ATG AAG-3′	18	57.38
